# Water Productivity Evaluation under Multi-GCM Projections of Climate Change in Oases of the Heihe River Basin, Northwest China

**DOI:** 10.3390/ijerph16101706

**Published:** 2019-05-15

**Authors:** Liu Liu, Zezhong Guo, Guanhua Huang, Ruotong Wang

**Affiliations:** 1 College of Water Resources and Civil Engineering, China Agricultural University, Beijing 100083, China; gzz1129921205@sina.com (Z.G.); ghuang@cau.edu.cn (G.H.); hy96980308@cau.edu.cn (R.W.); 2 Center for Agricultural Water Research in China, China Agricultural University, Beijing 100083, China

**Keywords:** climate change, spatio-temporal variation, crop growth, SWAP-EPIC model, Hexi Corridor, the Belt and Road

## Abstract

As the second largest inland river basin situated in the middle of the Hexi Corridor, Northwest China, the Heihe River basin (HRB) has been facing a severe water shortage problem, which seriously restricts its green and sustainable development. The evaluation of climate change impact on water productivity inferred by crop yield and actual evapotranspiration is of significant importance for water-saving in agricultural regions. In this study, the multi-model projections of climate change under the three Representative Concentration Pathways emission scenarios (RCP2.6, RCP4.5, RCP8.5) were used to drive an agro-hydrological model to evaluate the crop water productivity in the middle irrigated oases of the HRB from 2021–2050. Compared with the water productivity simulation based on field experiments during 2012–2015, the projected water productivity in the two typical agricultural areas (Gaotai and Ganzhou) both exhibited an increasing trend in the future 30 years, which was mainly attributed to the significant decrease of the crop water consumption. The water productivity in the Gaotai area under the three RCP scenarios during 2021–2050 increased by 9.2%, 14.3%, and 11.8%, while the water productivity increased by 15.4%, 21.6%, and 19.9% in the Ganzhou area, respectively. The findings can provide useful information on the Hexi Corridor and the Belt and Road to policy-makers and stakeholders for sustainable development of the water-ecosystem-economy system.

## 1. Introduction

Climate change has significant impacts on hydrological, biological, and ecological systems which are all closely related to water resources, causing sustainability concerns around the world. The Fifth Assessment Report (AR5) of the Intergovernmental Panel on Climate Change (IPCC) confirms that warming in the climate system is unequivocal, with many of the observed changes unprecedented over decades to millennia. Temperatures at the Earth’s surface in each of the last three decades has been successively warmer than that in any preceding decade since 1850 [1]. These facts indicate that future climate change mainly characterized by warming and tightly related with socio-economic development will be of significant global, national and regional importance. Understanding the evolution mechanism of climate change has thus become a priority area, both for research and for water management strategies. In the past 100 years, the increase of the land surface air temperature in China was greater than that of the whole world, especially in the inland regions of Northwest China with an increase >0.5 °C since the 1980s [2]. Compared with temperature, precipitation and its influencing factors are more complex and uncertain with no consistent trends in Northwest China, which cause much more vulnerability and sensitivity of agricultural water consumption and productivity to climate change [3].

In terms of the Representative Concentration Pathways emission scenarios (RCPs) according to the radiative forcing target level for 2100, general circulation models (GCMs) provide multiple global large-scale climate information for impact assessment, however, with a relatively coarse spatial resolution. Facing this problem, downscaling methods have been developed to generate higher-resolution climatic factors for regional studies based on GCM outputs [4,5]. The Statistical Downscaling Model (SDSM) developed by Wilby et al. [6,7,8] has been widely used to investigate the impacts of regional climate change on water resources. Fan et al. [9] demonstrated that the SDSM showed a high efficiency for simulating the air temperature in Northern China, indicating a significant trend of increasing air temperature in the future. Compared with the high accuracy of the air temperature, the simulated monthly precipitation by the SDSM exceeded the observed value [10]. Hao et al. [11] applied the SDSM to project the spatio-temporal characteristics of the future air temperatures and precipitation in the Hexi Corridor, implying that the daily maximum, minimum, and mean air temperature (T_max_, T_min_, T_mean_) all exhibited an increasing trend, while the precipitation showed significant regional variations, declining in the eastern and central parts and increasing in the western part. However, these previous studies have neglected the performance assessment on GCM selection in terms of specific study areas, which could cause great uncertainties in the regional impact evaluation of climate change [12,13]. To solve this problem, Wang et al. [14] firstly evaluated the 23 GCMs’ performances in the HRB, and then constructed the SDSM to provide the variation ranges of different climate variables simulated by multi-GCM under multiple scenarios, providing a reference for comparison between different GCMs and the concurrent issues related to downscaling. Climate change scenarios generated by Wang et al. [14] were used in this study.

As the second largest inland river basin in the arid region of Northwestern China, the HRB plays an important role for the production of commodity grains and the development of the Belt and Road initiative. Both the agricultural irrigation in the middle oasis and the fragile ecosystems in the downstream basin strongly depend on Heihe River water. However, the irrigation water diversion of the middle oasis accounts for more than 80% of the river runoff at Yingluoxia [15]. To restore the ecosystems in the downstream HRB, the Ecological Water Diversion Project (EWDP) has been applied since 2002. According to the EWDP, the river water allocated to the middle oasis is to be significantly reduced for increasing runoff discharge. Yet, the total water use is actually not reduced in the middle oasis [16] due to inappropriate land and water use and management [17,18]. More groundwater is exploited to supplement irrigation. This has resulted in a declining trend in groundwater levels [19,20] and the shrinkage of wetland and grassland areas [21] in the middle oasis. Therefore, how to optimize the water allocation ratio to the middle oasis and increase the agricultural water productivity becomes a challenge to the HRB [22].

Studies concerning hydrology, ecology, and economy have been accordingly conducted to reveal and synergize the complicated relationship of water-agriculture-ecology in the HRB. Kang et al. [23] demonstrated that water availability and crop production were likely to decrease in the future in response to increasing temperatures and fluctuating precipitation. Cao et al. [24] conducted a study on corn in the oasis irrigation areas of the Hexi Corridor indicating that the accumulated temperature in irrigated areas in the Hexi Corridor was significantly increased. The accumulated temperature during corn growth is a key factor affecting corn yield. As the climate in the irrigated areas warms, the corn yield in selected local areas has increased. The yield increases, from west to east, were 124%, 186%, and 301%, respectively, however, only one climate factor (temperature) was taken into consideration in this study. Zhao et al. [25] found that the air temperature in the HRB increased significantly from 1960 to 2009, which was closely correlated with the increasing yields of spring wheat, corn, and cotton, but higher pest population levels. Zhang et al. [26] investigated the responses of crop water use efficiency (WUE) to climate variables in the semi-arid area of Northern China during 1983–2010, suggesting that the difference of crop WUE in warm-dry environment and in warm-wet environment ranged from 29.0–55.5%. Changes in temperature and precipitation in the past three decades jointly enhanced crop WUE by 8.1–30.6%. Jiang et al. [17] developed a distributed agro-hydrological model to quantify the combined effects of weather, crop, soil and irrigation factors on irrigation performance and water productivity in the irrigated areas of the milled HRB, which implied that the water productivity was spatially varied and quite small due to excessive irrigation water use. Xu et al. [27] applied a distributed agro-hydrological model to access the irrigation water use in the major irrigation system of middle HRB, indicating that only 53% of total applied water was efficiently used, whereas deep percolation loss and canal conveyance loss accounted for 22% and 25% of the total applied water, respectively. It has been shown that since the late 1980s, the frequency and intensity of extreme hydrological-meteorological events have increased in Northwest China, which has caused serious adverse effects on local livelihood, such as agricultural production, water resources sustainable management, and food security, and has restricted the regional socio-economic development [28,29,30,31]. To sum up, this study aims to evaluate the impacts of climate change on water productivity in the irrigated oases of the middle HRB by: (1) using multi-GCM projections which could effectively reduce uncertainties from GCMs; (2) applying an agro-hydrological model to simulate the crop growth based on different climate change scenarios and crop reference evapotranspiration (*ET*_0_) scenarios derived from the Hargreaves equation adjusted by the Penman–Monteith equation; and (3) selecting two typical agricultural production areas to quantify variation patterns of the crop water productivity. Results obtained in this study will be helpful for synergic development of water security and agricultural production in arid inland regions adapt to climate change.

## 2. Materials and Methods

### 2.1. Study Region

**Figure 1:**
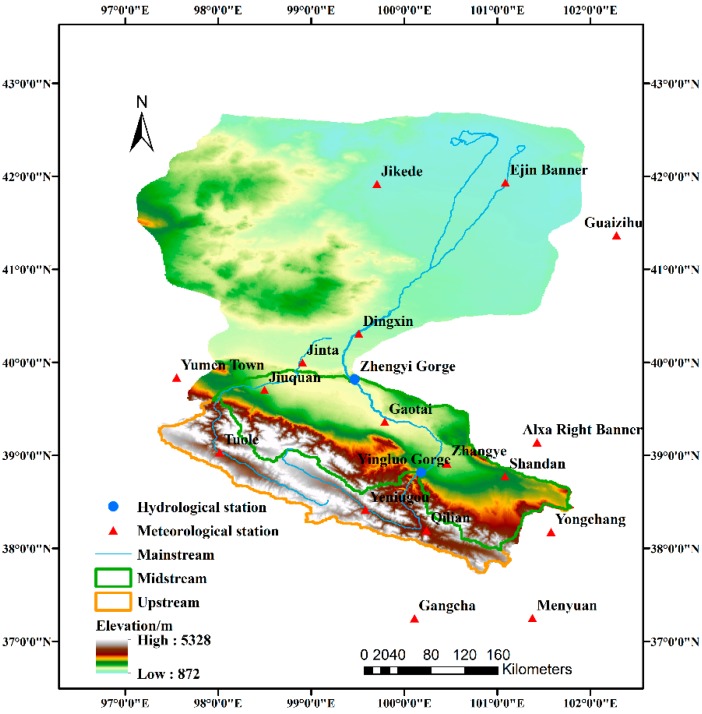
Sketch map of the Heihe River basin and gauging meteorological and hydrological stations.

The Heihe River basin as shown in Figure 1, bounded by longitudes 97°37′ and 102°06′ E and latitudes 37°44′ and 42°40′ N, covering an area of approximately 154,000 km^2^ [3]. The HRB is a typical inland river basin in the arid zone of Northwest China, which originates in the Qilian Mountain region and consists of three sections from south to north: the upstream area is from the Qilian Mountains to the Yingluoxia (outlets of the mountains), the midstream area runs from the Yingluoxia to Zhengyixia, and the downstream area occurs below the Zhengyixia and terminates in the Juyan Lakes (east and west branches, respectively). The study area has strong spatial heterogeneity with diverse ecosystems including an ice-snow zone, frozen soil, mountain forest, oasis, desert, and desert riparian forest [32,33,34]. The HRB is characterized by a continental climate. According to different regions, the average annual air temperature is from 2–3 °C in the upper HRB, from 6–8 °C in the middle HRB, and from 8 °C to 10 °C in the lower HRB. The mean annual precipitation is from 200–500 mm, from 120–200 mm, and below 50 mm in the upstream, midstream, and most downstream areas, respectively [35,36]. From southern mountain region to the Northern Gobi desert, the annual potential evapotranspiration varies from 500–4000 mm.

The HRB is also an important industrial and agricultural region in the western part of China, which covers predominantly arid and semi-arid areas with extremely fragile ecological conditions and a low level of adaptive capacity in terms of social living conditions. Significant climate variability in this region would cause more severe impacts on water resources, food production, and ecosystem health due to its poor ability to adapt to climate change security [37,38,39].

### 2.2. Data

The datasets consist of the observed and GCM-projected precipitation, T_mean,_ T_max_, and T_min_. The observed daily data at 17 meteorological gauging stations of the HRB (Figure 1) were taken from the China Meteorological Data Service Center. The 17 different gauging stations are located in areas with different climactic conditions. Given the reliability and integrity, the observed data from 1961–2010 were selected in this study. Considering the spatial variability between stations, missing data accounting for approximate 2% of the total were generated by establishing the linear regression between the stations with missing data and their nearest stations, which were characterized by the same climate conditions. Monthly and daily data from 23 GCMs corresponding to the observed climate variables were obtained from the phase 5 of the Coupled Model Intercomparison Project (CMIP5) archive (http://cmip-pcmdi.llnl.gov/cmip5/). The monthly data during 1961–2000 from 23 GCMs with different spatial resolutions were uniformly interpolated to the 2° × 2° spatial resolution to conduct the GCM performance evaluation, while the daily data were applied to generate the climate change scenarios by the statistical downscaling for the reference period (1976–2005) and the future period (2021–2050) under three RCPs (RCP2.6, RCP4.5, and RCP8.5) [40]. The RCPs are named depending on radiative forcing target level for 2100. RCP2.6 is defined that the peak in radiative forcing increased to 3 W/m^2^ before 2100 and then declines to 2.6 W/m^2^ (around 490 ppm CO_2_ eq.) by 2100. The definition of the RCP 4.5 and RCP 8.5 are described as the stabilization without overshoot pathway to 4.5 W/m^2^ (around 650 ppm CO_2_ eq.) at stabilization after 2100 and the rising radiative forcing pathway leading to 8.5 W/m^2^ (around 1370 ppm CO_2_ eq) by 2100 [41]. Detailed information of the 23 GCMs referred to Wang et al. [14].

### 2.3. Methods

#### 2.3.1. Multi-GCM Performance Evaluation

The performance of the 23 GCMs simulations of monthly precipitation, T_mean,_ T_max_, and T_min_ were evaluated by the score-based method proposed by Fu et al. [42]. A multi-criteria rank score (*RS*) value of 0–10 was calculated for each individual assessment criterion as:

(1) $$RS_{i}=\frac{X_{max}-X_{i}}{X_{max}-X_{min}}\times 10$$

where $X_{i}$ is the relative error or relationship statistic between the GCM output and observed for the *i*th GCM. *X_max_* and *X_min_* are the maximum and minimum of relative error, respectively. High *RS* values indicate high performance of GCMs. The total *RS* for each GCM for a specific climate variable was obtained by summing all *RS* for all criteria used. In this study, a weight of 0.5 was allocated to the criteria including trend analysis, trend magnitude, the first two leading modes of each empirical orthogonal function (EOF1 and EOF2), and two PDF (probability density function) criteria, namely *BS* (brier score) and *S*_score_ (significance score), while the other assessment criteria had a 1.0 weight in the summation. This total *RS* was then used to rank the GCMs for all climate variables [42]. Eleven statistics used in this study are shown in Table 1.

The score-based method has already been used for the multi-GCM performance evaluation in different regions [43,44]. In this study, the performances of the 23 GCMs were evaluated on the basis of the observed monthly precipitation and mean/maximum/minimum air temperatures during 1961–2000 from the 17 meteorological stations and the simulated monthly data from GCMs for the same period.

#### 2.3.2. The SWAP-EPIC Model

SWAP-EPIC was a one-dimensional (1-D) physical-based agro-hydrological model that could simulate soil water flow, solute and heat transport as well as crop growth on a field scale and daily time-step. It was proposed by Xu et al. [27] through coupling the SWAP (Soil-Water-Atmosphere-Plant) model [45] and the EPIC (Environmental Policy Integrated Climate) crop growth module [46], which is a less demanding data input for crop growth modeling and more suitable for regional application. The soil water flow was described based on the 1-D Richards equation for vertical flow. Soil hydraulic properties were described using the van Genuchten [47] and Mualem [48] functions. The solute transport and heat transfer were described with the form of convection-dispersion equation and the conduction equation, respectively. The finite-difference solution scheme was applied for both solving the partial differential equations of soil water flow, solute transport, and heat conductance. The modules of soil water flow and crop growth were adopted for the current study. A brief description of the SWAP-EPIC model is provided in this paper, while more detailed theory can be found in [17,27,45].

The top boundary condition can be determined by the actual evaporation and transpiration rates and the irrigation and precipitation fluxes. Reference crop evapotranspiration (*ET*_0_), estimated by the Penman–Monteith equation [49] by using daily meteorological data (radiation, wind speed, vapor pressure, and temperature), was used to calculate potential evapotranspiration (*ET_p_*), which was then partitioned into potential soil evaporation and potential crop transpiration based upon the leaf area index [50]. Actual evaporation and transpiration rates were respectively obtained as a function of the available soil water in the surface soil and the root zone. Crop growth and yield were calculated using the modified EPIC crop growth model [46]. This module described the crop growth based on accumulated temperature. It can simulate the leaf area development, light interception, and the conversion of intercepted light into biomass and yield together with effects of temperature, water and salt stress.

The GIS-based SWAP-EPIC was developed through a close coupling of SWAP-EPIC and ArcInfo GIS, using VBA programs in GIS environment by Jiang et al. [17]. Thus, the SWAP-EPIC was extended to be used in a distributed manner for regional modeling. The distributed modeling was conducted by identifying a heterogeneous area as an assemblage of individual simulation units with homogenous soil-water-plant-weather representation. Independent runs of SWAP-EPIC model can simulate soil water balance and crop growth and yield in each simulated unit. The detailed description about GIS-based SWAP-EPIC model was given by Jiang et al. [17].

#### 2.3.3. Statistical Downscaling for Climate Change Scenarios

The SDSM developed by Wilby et al. [6,7,8] was adopted to generate climate change scenarios in this study. The SDSM screens the predictors closely related to the predictands and builds an empirical statistical relationship between the predictands (climate variables of the observed meteorological stations) and the predictors (ERA-40 reanalysis data) based on multiple linear regression. In summary, the SDSM assumes that the empirical statistical relationship is constant in the case of future changes in climatic conditions.

Screening downscaling predictors is key to the SDSM, and largely determines the results of downscaling and future climate scenarios [7]. The selection of predictors is an iterative process, based on the results of seasonal correlation analysis, partial correlation analysis, and scatterplots. Furthermore, seasonal correlation analysis is conducted by investigating the percentage of variance explained by specific predictand–predictor pairs and by judiciously concerning the most appropriate combination of predictors for a given season and predictand. Partial correlation analysis is used to investigate inter-variable correlations. These statistics help to identify the amount of explanatory power that is unique to each predictor.

In this study, calibration processes of temperature and precipitation were determined as unconditional and conditional processes, respectively [7]. The SDSM was calibrated by artificially inflating the variance inflation (*VIF*) and bias correction (*BC*) of downscaled series based on their change ranges to accord better with daily observations. Furthermore, 0 < *VIF* < 10 indicates no correlation; 10 < *VIF* < 100 indicates a moderate correlation; *VIF* ≥ 100 indicates a high correlation. The *VIF* default value is 12. The value of *BC* ranges from 0–2. The BC default value is 1.0, which indicates no bias correction [6].

## 3. Results and Discussion

### 3.1. Multi-GCM Performance Evaluation

**Figure 2:**
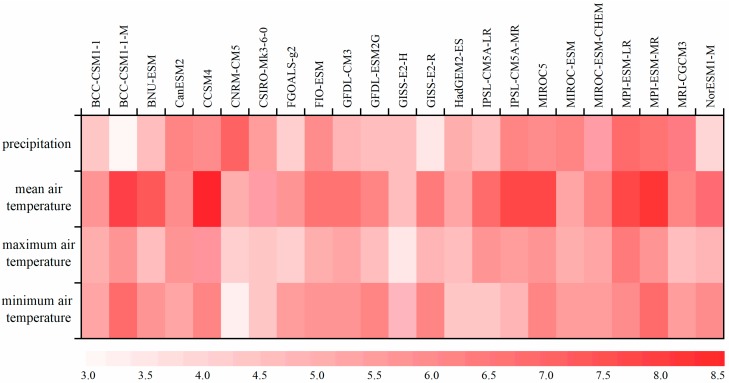
Performances of 23 GCMs for the precipitation, T_mean,_ T_max_, and T_min._

As shown in Figure 2, the *RS* values for the precipitation, T_mean,_ T_max_, and T_min_ showed significant differences in the 23 GCMs. The GCM showing the best simulation effect of precipitation was the CNRM-CM5 (*RS* = 7.15), while the GCMs displaying best performance of the T_mean,_ T_max_, and T_min_ were the CCSM4 (*RS* = 8.55), MPI-ESM-LR (*RS* = 6.33), and BCC-CSM1-1-M (*RS* = 6.93), respectively. Compared with significant differences of precipitation performances in 23 GCMs (*RS* values ranged from 2.99–7.15), GCM performances of air temperatures showed to be relatively consistent, of which the *RS* values were all above 5.0 for the T_mean_, while the *RS* values for the T_max_ and T_min_ ranged from 3.43–6.33 and from 3.32–6.93, respectively. In this study, the MPI-ESM-MR (*RS* = 8.28) was finally selected to project the future T_mean_ instead of the CCSM4 with the highest *RS* value (*RS* = 8.55) due to its lacunae in long-term time series of the T_mean_. To sum up, CNRM-CM5, MPI-ESM-MR, MPI-ESM-LR, and BCC-CSM1-1-M were used to project the future precipitation, T_mean,_ T_max_, and T_min_ in the HRB, respectively.

### 3.2. Multi-GCM Projections of Climate Change

Based on the downscaling framework proposed by Guo [51] and Wang et al. [14], the climate change scenarios of the precipitation, T_mean,_ T_max_ and T_min_ during 1976-2005 (baseline period) and 2021–2050 (future period) under the three RCP scenarios (RCP2.6, RCP4.5, and RCP8.5) in the HRB were generated.

**Figure 3:**
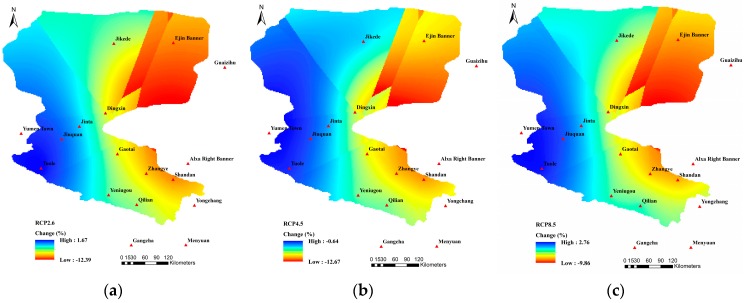
Spatial variation patterns of the mean annual precipitation under (**a**) RCP2.6; (**b**) RCP4.5; and (**c**) RCP8.5.

As shown in Figure 3 and Table 2, compared with the precipitation during the baseline period at the 17 stations (excepting the Tuole and Qilian stations), the mean annual precipitation over the next 30 years showed a decreasing trend under all three RCP scenarios. The mean annual precipitation of the Tuole station had an increasing trend under all three RCP scenarios, while that of the Qilian station decreased under RCP2.6, but increased under RCP4.5 and RCP8.5. Among the 15 stations with decreasing precipitation in the future, the decreasing magnitude of the future precipitation in Jikede, Guazihu, Yumen Town, Alxa Right Banner, Yongchang, and Menyuan declined with the concentration increase of RCP, i.e., RCP2.6 > RCP4.5 > RCP8.5, while there was no clear pattern for the amount of decrease in precipitation at the other stations. From the whole basin perspective, the precipitation of stations in the upper reaches of the HRB under three future climate scenarios decreased only slightly or even increased (e.g., Qilian area), while the precipitation in the border areas of the middle and lower reaches decreased by more than 15%. According to the Table 1, the mean annual precipitation in the future period under the three RCP scenarios would all decline with the decrease being the most obvious under the RCP4.5 scenario, up to 5.22%.

**Figure 4:**
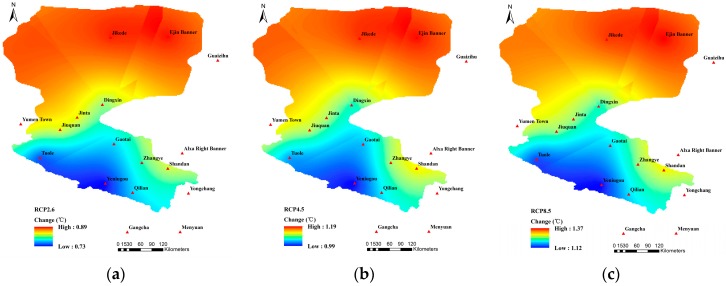
Spatial variation patterns of the T_mean_ under (**a**) RCP2.6; (**b**) RCP4.5; and (**c**) RCP8.5.

As shown in Figure 4, compared with the reference period, the T_mean_ during the future period under RCP2.6, RCP4.5, and RCP8.5 all showed an increasing tendency, which was more significant as the concentration of RCP increased, i.e., RCP8.5 > RCP4.5 > RCP2.6. The T_mean_ increased more than 1 °C under both RCP4.5 and RCP 8.5 scenarios while, under the RCP2.6 scenario, the increase of the T_mean_ exhibited a relative lower value (<1 °C). From the regional perspective, the greatest temperature increase located in Ejin Banner, Jikede, Shandan, Yumen Town, and Qilian. Temperature increase was comparatively less in Gaotai, Yongchang, and Gangcha. The T_mean_ in the southern mountainous areas of the HRB was relatively low, while the temperature in the northern desert was comparatively high, with the T_mean_ increasing gradually from the southwest to the northeast. As shown in Table 1, compared with the reference period, the annual T_mean_ during the next 30 years increased by 0.84 °C, 1.14 °C, and 1.28 °C under the three scenarios, respectively, with the most significant temperature rise being in areas located in the lower reaches of the HRB, including Jikede, Ejin Banner, and Guaizihu.

**Figure 5:**
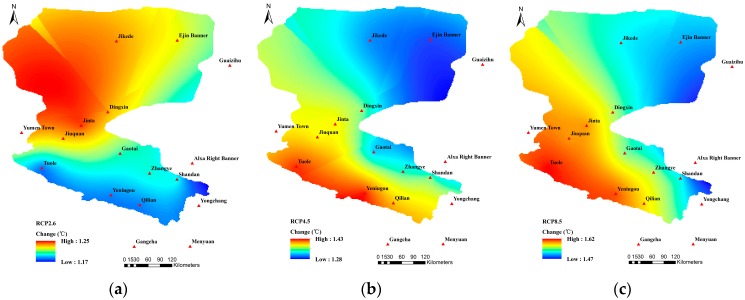
Spatial variation patterns of the T_max_ under (**a**) RCP2.6; (**b**) RCP4.5; and (**c**) RCP8.5.

As shown in Figure 5, under the three RCP scenarios, the mean annual T_max_ of all the stations in the HRB showed an upward trend with a considerable rise. The highest temperature rise was in the Qilian area, where the T_max_ increased by 1.69 °C under the RCP 8.5 scenario. From the perspective of the entire basin, Table 1 indicated that the T_max_ under the three RCP scenarios increased by 1.23 °C, 1.35 °C, and 1.55 °C, respectively, which was more remarkable than the increase of the mean annual air temperature.

**Figure 6:**
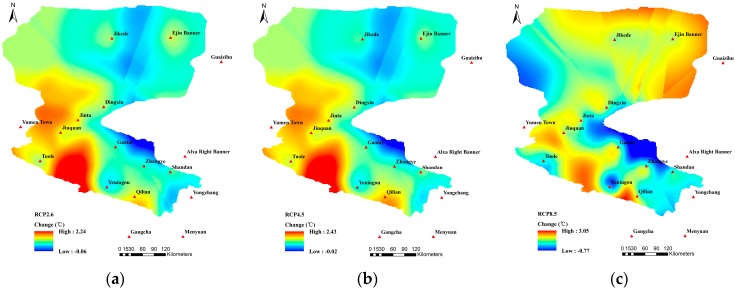
Spatial variation patterns of the T_min_ under (**a**) RCP2.6; (**b**) RCP4.5; and (**c**) RCP8.5.

Different from higher increases of the T_max_ than those of the T_min_ under the RCP 2.6 and RCP 4.5 scenarios, the increases of the T_min_ at some stations were higher than those of the T_max_ under the RCP8.5 scenario, including Yumen Town (1.76 °C), Jinta (1.84 °C), and Jiuquan (1.75 °C). Areas with relatively small increases of the T_min_ included Jikede, Yeniugou, Yongchang, Gangcha, and Mengyuan. As shown in Figure 6, the highest increase of the T_min_ situated in the southwestern mountainous area. However, in contrast to the spatial variation patterns of the T_mean_ and T_max_, T_min_ rose the most in the middle reaches. Referring to Table 1, compared with the reference period, the T_min_ during the future period under RCP2.6, RCP4.5, and RCP8.5 all increased by 1.08 °C, 1.18 °C, and 1.68 °C, respectively.

### 3.3. Preparation for SWAP-EPIC

The Gaotai and Ganzhou irrigated areas situated in the middle reaches of the HRB were selected as the typical oasis agricultural areas in this study for crop water productivity evaluation under climate change. As the national maize seed production base in these two areas, the water consumption of maize is of significant importance for water saving in agriculture. In this study, maize was chosen as the crop to be simulated in the SWAP-EPIC model and the actual irrigation scheduling used in the field experiment in 2012 was adopted. Under the three future climate scenarios, the irrigation schedule remained unchanged while the climate variables were changed to analyze impacts of climate change on the water productivity of maize in the oasis agricultural areas.

Details about the inputs including historical data and future scenarios to drive the SWAP-EPIC model could be found in Guo [51]. The future wind speeds in both Gaotai and Ganzhou showed a declining trend, and the decrease of wind speeds under RCP scenarios was RCP4.5 > RCP8.5 > RCP2.6 and RCP8.5 > RCP4.5 > RCP2.6, respectively. The solar radiation *R_S_* and actual water vapor pressure $e_{a}$ under future climate scenarios were deduced by estimating missing meteorological data using the method proposed by FAO56 [52]. However, the sunshine duration required by the SWAP-EPIC model to calculate *ET*_0_ using the Penman–Monteith equation could not be obtained directly from the projected climate scenarios in this study. Instead, the modified Hargreaves equation requiring less inputs was used to calculate based on the linear regression analysis with the results of Penman–Monteith equation. The empirical coefficient *K*, the exponential coefficient *n*, and the temperature offset *T_off_* of the original Hargreaves equation [53] could not be directly applied to the HRB, which were needed to be calibrated. Daily T_min_, T_max_, and solar radiation in Gaotai and Ganzhou from 1961–2000 were used as the independent variables, while the daily *ET*_0_ calculated by the Penman–Monteith equation was taken as the dependent variable, the original parameters of the Hargreaves equation *K* = 0.0023, *n* = 0.5, and *T_off_* = 17.8 as the initial values, the linear regression analysis was conducted to determine parameters for the Gaotai and Ganzhou areas. Modified Hargreaves equations for the Gaotai and Ganzhou areas are as follows, respectively:

(2) $$ET_{0}=0.001{\left(T_{max}-T_{min}\right)}^{0.336}\left(T_{mean}+23.507\right)R_{a}$$

(3) $$ET_{0}=0.001{\left(T_{max}-T_{min}\right)}^{0.410}\left(T_{mean}+23.489\right)R_{a},$$

To further validate the effect of the modified Hargreaves equation, comparison between results calculate by the Penman–Monteith equation and the modified Hargreaves equation was conducted based on daily meteorological data of Gaotai and Ganzhou from 2001 to 2011. *R*^2^ of the calculated *ET*_0_ by the two equations were 0.868 and 0.884, respectively, which indicated that the modified Hargreaves equation could be used to calculate the *ET*_0_ under the three future climate scenarios of RCP2.6, RCP4.5, and RCP8.5.

### 3.4. Water Productivity Evaluation

**Figure 7:**
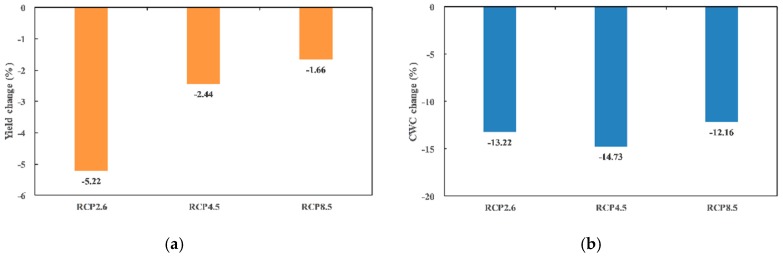
Changes of the mean annual (**a**) maize yield and (**b**) CWC in the Gaotai area under RCP2.6, RCP4.5, and RCP8.5.

In this study, the maize growth of the Gaotai and Ganzhou irrigated areas was modeled by the constructed SWAP-EPIC model under the three RCP scenarios to derive the crop yield and *ET_a_* defined as crop water consumption (CWC) [54] in this study. The calibrated and validated parameters of the SWAP-EPIC model refer to Jiang et al. [17] and Jiang [32] for the oases of the middle HRB were used in this study. As shown in Figure 7, compared with the field experiment period of 2012–2015, the mean annual yield of maize in the Gaotai area under the three RCP scenarios all showed a decreasing trend. Meanwhile, the decreasing magnitude of the future mean annual yield in Gaotai area declined with the CO_2_ concentration increase of RCP, i.e., RCP2.6 > RCP4.5 > RCP8.5. Although temperatures were projected to keep rising in the next 30 years, the mean annual CWC exhibited a decreasing trend due to unchanged irrigation schedule and projected decreases of precipitation. The decreases of CWC under different RCP scenarios all exceeded −12%, with the largest decline being −14.73% under the RCP4.5 scenario. In addition, Xu et al. [27,55] indicated that CO_2_ concentration was of great importance for the simulation of crop yield by influencing the photosynthesis, plant respiration and evapotranspiration in the SWAP-EPIC model, especially in arid inland regions of Northwest China. In this study, from the perspective of elevated CO_2_ concentration defined in the radiative forcing target level of RCP2.6, RCP4.5, and RCP8.5, potential effects of CO_2_ enrichment might be of great importance on the maize yield and CWC in the irrigated areas of the HRB basin.

Table 3 showed the inter-decadal maize yields under the three RCP scenarios in the Gaotai area. The mean annual maize yield simulated was 12,806 kg/ha from 2012–2015, while the mean inter-decadal maize yields under the three future climate scenarios simulated from 2021–2030 and from 2031–2040 were both lower than the historical simulation results, which caused the overall decrease of the maize yield in the future 30 years. Although there was a decrease of the maize yield in the future, which was compared with the yield in the historical period, the future inter-decadal maize yield in the Gaotai area under the scenarios of RCP2.6 and RCP8.5 gradually increased with time while, under the RCP4.5 scenario, the inter-decadal maize yield (12,204 kg/ha) in the early period was higher than that (11,924 kg/ha) in the middle period.

Table 4 showed the inter-decadal CWC of maize under the three RCP scenarios in the Gaotai area. The CWC of maize from 2012–2015 was 677.7 mm, while under the RCP2.6, RCP4.5, and RCP8.5 scenarios, the inter-decadal CWC was lower than the historical simulation results. Similar to the simulation results of the inter-decadal maize yield in Gaotai, the inter-decadal CWC of maize gradually increased with time under the RCP2.6 and RCP8.5 scenarios, while under the RCP4.5 scenario, the inter-decadal CWC of maize (556.4 mm) in the middle period was lower than that in the early and later periods. Inconsistency between decreased maize yield/CWC and projected increases in air temperatures might be attributed to the fluctuating decreases of precipitation under the three RCP scenarios (Table 2) and unchanged irrigation schedule in this study, implying the significant importance of precipitation-temperature-irrigation synergy effect on the crop yield in the middle HRB, which was also demonstrated by Kang et al. [23] and Zhang et al. [26].

**Figure 8:**
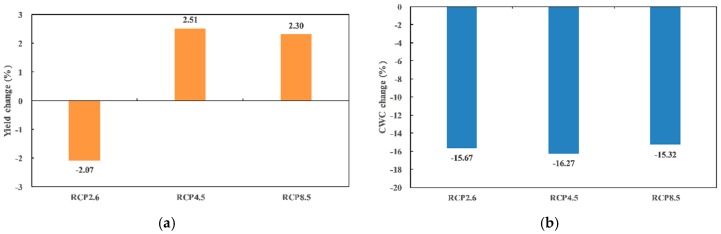
Changes of the mean annual (**a**) maize yield and (**b**) CWC in the Ganzhou area under the three RCP scenarios.

Figure 8 shows changes of the mean annual maize yield and CWC under the three RCP scenarios in the Ganzhou area compared with simulation results from 2012–2015. Different from the increases of the mean annual maize yield with the rates of 2.51% and 2.3% under RCP4.5 and RCP 8.5 scenarios, the mean annual maize yield under the RCP2.6 scenario exhibited a decreasing trend. Similar with changing patterns of the CWC in the Gaotai area, the mean annual CWC in the Ganzhou area showed a declining trend under all three RCP scenarios with the highest decreasing rates of RCP4.5 (−16.27%) and the lowest decreasing rates of RCP8.5 (−15.32%).

The inter-decadal mean maize yields under the three RCP scenarios in the Ganzhou area were shown in Table 5. The mean maize yield simulated in Ganzhou was 11,504 kg/ha from 2012–2015, and the inter-decadal maize yields under the three future climate scenarios gradually increased with time. During the early period (2021–2030) under the three RCP scenarios, the maize yield was lower than the historical simulation results, while the maize yields in the middle and later periods (2031–2041 and 2041–2050) were higher than the historical simulation results.

Table 6 shows the inter-decadal CWC of maize in the Ganzhou area under the three future climate scenarios. Similar to the simulation results of the inter-decadal CWC of maize in the Gaotai area, the mean CWC of maize in Ganzhou was 686.3 mm from 2012–2015, and the inter-decadal mean CWC of maize under all future climate scenarios of RCP2.6, RCP4.5, and RCP8.5 was lower than that of the reference period. The inter-decadal mean CWC of maize gradually increased with time under the RCP2.6 and RCP8.5 scenarios, while under the RCP4.5 scenario, the inter-decadal CWC of maize (559.5 mm) in the middle period was lower than that in the early and later periods.

Water productivity (WP) is used to quantify the crop yield per unit water use [56]. WP values change with the spatial scale and with the terms used to estimate water use (soil evaporation, deep percolation and water conveyance operational losses). Thus, various approaches to define WP have been used aiming at providing useful indicators for evaluation of irrigation performance and improving irrigation water-saving management practices [17,57,58,59,60]. In this study, the regional water productivity WP (kg m^−3^) for the Gaotai and Ganzhou areas was described as follows:

(4) $$\mathrm{WP}=\frac{\sum_{i}^{n}Y_{i}\times A_{su,i}}{\sum_{i}^{n}W_{i}\times A_{su,i}}$$

where *Y* is the crop yield (kg ha^–1^), *W* is the amount of crop water consumption *ET_a_* (m^3^ ha^−1^), *A_su_* is the area of the considered simulation unit (ha), and the subscript *i* denotes serial number of the simulation unit, while *n* is the total number of simulation units.

**Figure 9:**
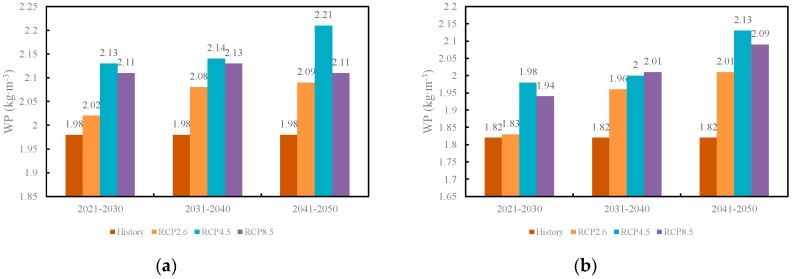
Decadal changes of the maize WP in the (**a**) Gaotai area and (**b**) Ganzhou area under the three RCP scenarios.

Figure 9 illustrated the inter-decadal water productivities under the historical period and three future climate scenarios in the Gaotai and Ganzhou areas. Driven by the projected climate change scenarios including the increases of the T_mean_, T_max_, and T_min_, and the decreases of the wind speed and precipitation, the water productivities displayed an increase under all three RCP scenarios. The water productivities during the next three decades under all RCP scenarios were greater than those of the historical period from 2012–2015. Interestingly, the WP reached the greatest in both Gaotai and Ganzhou areas under the medium emission scenario (RCP4.5) instead of the extreme emission scenario (RCP8.5) with the most significant temperature rising, implying that water, heat, and carbon projected under the RCP4.5 scenario in the middle irrigated areas of the HRB synergized the best for the crop growth. As time went on, the WP became higher under all RCP scenarios, which was consistent with the increase of temperature.

## 4. Conclusions

In this study, the multi-GCM projected climate change under the scenario of RCP2.6, RCP4.5, and RCP8.5 coupled with an agro-hydrological model SWP-EPIC was proposed to evaluate the water productivity of maize in the middle irrigated regions of the Heihe River basin from 2021–2050.

Compared with the reference period, the result show that precipitation was predicted a decrease under the future climate scenarios, while the T_mean_, T_max_, and T_min_ all exhibited increasing trends. The increase of the T_max_ was greater than that of the T_mean_ and T_min_. Driven by the multi-GCM projections of climate change, the maize growth in the Gaotai and Ganzhou areas was simulated to obtain the yield, CWC, and WP. Compared with simulation results based on field experiments from 2012–2015, the WP in the two typical agricultural areas both exhibited an increasing trend, which was mainly attributed to decrease of crop water consumption. The water productivity under three RCP scenarios in the Gaotai area during 2021–2050 increased by 9.2%, 14.3%, and 11.8%, and which increased by 15.4%, 21.6%, 19.9% in the Ganzhou area, respectively, most of which were larger than 2 kg/m^3^, indicating that water-heat-carbon projected under the RCP4.5 scenario in the middle irrigated areas of the HRB synergized the best for maize growth. Although the irrigation schedule in this study was unchanged, implications of the relevant water saving practices for the irrigated areas of the middle HRB could also be obtained from the perspective of the increase of efficiency in the process of field water application. The specific suggestions emphatically include: (1) improving traditional irrigation methods and concepts of field water use (using the modernized basin, prompting the water-saving education for farmers); (2) implementing water-saving irrigation technologies and equipment in areas with higher income or powerful financial support. The findings can provide useful information on the Hexi Corridor and the Belt and Road to policy-makers and stakeholders for sustainable development of the water-ecosystem-economy system.

## Figures and Tables

**Table 1:** Statistics of climate variables used for GCM performance assessment.

Statistics of Climate Variables	Methods	Weights
Mean	Relative Error (%)	1.0
Standard deviation	Relative Error (%)	1.0
Temporal variation	NRMSE	1.0
Monthly distribution (Annual cycle)	Correlation Coefficient	1.0
Spatial distribution	Correlation Coefficient	1.0
Trend and its magnitude	Mann-Kendall test Z	0.5
	Mann-Kendall test β	0.5
Space-time variability	EOF 1	0.5
	EOF 2	0.5
Probability density functions (PDFs)	*BS*	0.5
	*S_score_*	0.5

**Table 2:** Changes of the mean annual precipitation and air temperatures during 2021–2050 under the three RCP scenarios.

Change	RCP2.6	RCP4.5	RCP8.5
Precipitation/%	−4.57	−5.22	−2.40
T_mean_/°C	0.84	1.14	1.28
T_max_/°C	1.23	1.35	1.55
T_min_/°C	1.08	1.18	1.68

**Table 3:** Decadal changes of the maize yield in the Gaotai area under the three RCP scenarios (unit: kg/ha).

Time	RCP2.6	RCP4.5	RCP8.5
2012–2015	12,806	12,806	12,806
2021–2030	11,431	12,204	12,108
2031–2040	12,106	11,924	12,398
2041–2050	12,876	13,352	13,275

**Table 4:** Decadal changes of the maize CWC in the Gaotai area under the three RCP scenarios (unit: mm).

Time	RCP2.6	RCP4.5	RCP8.5
2012–2015	677.7	677.7	677.7
2021–2030	565.3	573.1	573.4
2031–2040	582.8	556.4	582.1
2041–2050	616.2	604.2	630.4

**Table 5:** Decadal changes of the maize yield in the Ganzhou area under the three RCP scenarios (unit: kg/ha).

Time	RCP2.6	RCP4.5	RCP8.5
2012–2015	11,504	11,504	11,504
2021–2030	10,148	11,141	10,642
2031–2040	11,275	11,196	11,522
2041–2050	121,073	12,727	12,818

**Table 6:** Decadal changes of the maize CWC in the Ganzhou area under the three RCP scenarios (unit: mm).

Time	RCP2.6	RCP4.5	RCP8.5
2012–2015	686.3	686.3	686.3
2021–2030	553.2	562.1	548.4
2031–2040	575.3	559.5	572.9
2041–2050	601.2	596.6	614.7
